# Immunological Methods for Treatment of Vulvovaginal Infections in the Preconception Period

**DOI:** 10.25122/jml-2019-0068

**Published:** 2019

**Authors:** Olga S. Alyautdina, Elena V. Esina

**Affiliations:** 1. Department of Obstetrics and Gynecology, Sechenov First Moscow State Medical University, Moscow, Russian Federation; 2. MedSwiss Medical Center, Moscow, Russian Federation

**Keywords:** autolymphocyte therapy, immunotherapy, vulvovaginitis, pelvic inflammatory diseases, infertility, IALT - intravaginal autolymphocyte therapy, MALT - mucosal-associated lymphoid, PCR - polymerase chain reaction, STIs - sexually transmitted infections, tissue

## Abstract

Traditional therapy and extensive use of medications and intravaginal autolymphocyte therapy show different results of the treatment of vulvovaginal infections. The purpose of the article was to explore safe and highly effective methods to treat vulvovaginal infections and diseases of the pelvic organs.

The standard clinical and laboratory screening of 70 patients of reproductive age was carried out to diagnose the diseases of the reproductive tract. The screening included the description of quantitative and qualitative characteristics of vaginal discharge, examining the mucous covering of the vulva and vagina, microscopic examination of Gram-stained vaginal swabs, endocervical cultures, and diagnosis of sexually transmitted infections using polymerase chain reaction. Intravaginal autolymphocyte therapy was used together with traditionally-accepted treatment schemes (etiotropic antibacterial and antifungal therapy) in the treatment of the main group (40 patients). Traditional treatment methods depending on the etiology of the development of infection were used in the control group (30 patients). The IgM, IgA, and IgG levels were also observed because of the possibility of causing embryo rejection. This study shows that in case of relapsing vulvovaginitis and mixed infections accompanied by disorders of the immune system at different levels, the use of intravaginal autolymphocyte therapy in a comprehensive therapy can be assessed as advisable and pathogenetically substantiated.

## Introduction

The incidence of inflammatory diseases of the reproductive organs in Russia accounts for 28-34% of the overall gynecological pathology [1], whereas the general level of prevalence of sexually transmitted infections (STIs) over the last years amounted to 37.5% [2]. In the development of disorders of the female reproductive system, particular attention is drawn to the role of infectious diseases, which lead to infertility and early termination of pregnancy [3,4]. Practically, all vaginal microflora, except for Bifidobacteria and Lactobacillus, may be a reason for the development of inflammatory responses, especially urogenital STIs [5]. The pathogenesis of any inflammatory process on mucous membranes is evolving according to an overall pattern [1]. The initial stage includes the adhesion of the pathogen to the epithelium, after which a process of colonization of the mucous membranes starts. After pathogens pass the local physiological barrier, a generalized infection develops. If local protective mechanisms work effectively, the infectious process can stop at any stage.

These protective mechanisms include the most complex biological barrier systems formed during evolution. The primary natural protection barrier is the closed state of the vulvar cleft alongside the tone of the perineal muscular frame, which isolates the vagina from external influences. The next physiological barrier is the vagina itself. Firstly, its ability of self-purification as a result of desquamation and cytolysis of the epithelial cells of the mucous membrane, which depends on hormones in different cycle phases. Secondly, glycogen is accumulated in the mucous membrane cells under the influence of estrogens, and the adhesion of Lactobacilli is happening, taking part in the breakdown of glycogen, releasing lactic acid and moving the pH towards higher acidity (3.8–4.5). Such a level of acidity of vaginal secretions is considered optimal for the functioning of vaginal microflora and inhibiting the growth of opportunistic and pathogenic microorganisms [6]. Defense mechanisms of lactobacilli are based not only on by their antagonistic activity and adhesion properties but also on their ability to synthesize antibiotic substances – hydrogen peroxide, lysozymes, lactocidin, and acidophilus. The most important protective barriers of the vagina include local factors of immune defense and is represented by the mucosal-associated lymphoid tissue (MALT), which ensures fast localization of the infectious agent; consequently, the infectious process involves a minimal inflammatory response. This is the result of a comprehensive action of non-specific mechanisms, in particular, the phagocytic system and specific response of the immune system, namely the production of antibodies [7].

The primary functions of macrophages, which are part of the phagocytic system, are phagocytosis and killing of microorganisms. Neutrophils represent the first line of non-specific defense on the surface epithelium. They are the first to be at the site of inflammation or infectious process. Hence, their activity directly impacts the elimination of a harmful agent [4]; the second line of defense of the blood-tissue interface is represented by the macrophage system. As soon as an infectious agent penetrates the epithelial barrier to the subepithelial connective tissue, it starts to interact with macrophages, which have high phagocytic activity, functional mobility, and a unique ability to synthesize toxic metabolites of oxygen and powerful enzymes that are able to take part in hydrolysis. The contact of the pathogen with the macrophages’ receptors induces the production and secretion of cytokines with an anti-inflammatory effect, which provides an early inflammatory response [5].

Interferons (IFNs)-α, -β, -γ belong to a group of low-molecular glycoproteins synthesized by leukocytes and MALT cells, particularly lymphocytes and fibroblasts, in response to antigen induction. In this process, they form the first protective barrier in case of an infection of viral etiology, ensuring the resistance of cells to a pathogen much earlier than the specific protective responses occur. Disorders in the IFNs system lead to a failure of the majority of immune elements – antibacterial, antiviral, antiprotozoal, and so forth [8-10]. One of the critical effector systems of the local protective barrier is the immunoglobulin profile of the vaginal environment, including secretory IgA (sIgA), whose role is crucial in the non-specific anti-inflammatory defense of mucous membranes. Its significant effects include the prevention of adhesion of pathogenic microflora to the epithelium of the blood-tissue interface by forming intramembranous immune complexes that directly prevent the adhesion of pathogens to epithelium cells and thoroughly neutralize their activity on the biological level [11-13]. Intraepithelial lymphocytes are the major Ig-producers. Vaginal secretion is a biological liquid with a high concentration of IgG, which may be present both locally and on the systemic level in the conditions of a transudative mechanism [14].

The cervix is the third natural protective barrier in the female reproductive system, which prevents the penetration of infectious agents into the uterine cavity. The protective mechanism of the cervix barrier is formed by the narrow cervical channel covered by external and internal orifices and determined by the level of secretion of mucus by the columnar epithelium, concentration of lysozyme, and slgA in the mucus. Besides, the cervical canal has a local independent system of anti-bodies formation against some viral and bacterial pathogens [15]. Resistance of pelvic organs to infectious agents is provided by the cyclic desquamation of the functional layer of endometrium during menstruation, oxidizing and restorative, antibiotic processes in the epithelial cells of the uterus, peculiarities of peristalsis of the fallopian tubes and oscillating movement of the ciliated epithelium towards the uterus cavity, presence of germinal epithelium covering the ovaries, elements of immunological defense of the peritoneal fluid [6].

When protective mechanisms are breached on any level, an infectious process evolves, which causes specific morphological and functional changes in the female reproductive organs, leading to a pathological afference to the parts of the central nervous system that regulate the hypothalamus-pituitary-ovarian system, which inevitably results in functional disorders. Disruptions in the immune defense in the course of inflammatory diseases of the pelvic organs are manifested through the activity inhibition of the system of IFNs, natural killers and macrophages, as well as by inhibition of the cell element of immune defense (disbalance of T-cells and polyclonal stimulation of B cells) and an increase of the count of all types of Ig. Besides the impact on the immunity on a systemic level, pathogenic agents can cause severe changes locally, which are represented by an increase in the number of T cells, NK-cells, and macrophages. In the course of this, the count of classic СD16+ Nк-cells is rising in the endometrium, while in the decidual tissue that is amidst the infectious process, the number of activated cytotoxic NK-cells (CD57+), which produce embryotoxic cytokines, is increasing.

Furthermore, the number of cells responsible for the synthesis of the transforming growth factor beta-2 (TGF-β2), which has an immunosuppressing effect, is dropping [16, 17]. The macrophages that underwent activation can synthesize nitrogen oxide, toxic to trophoblast cells. Endometrial cells of healthy women produce type 2 T-helper (Th2) cytokines – interleukins 4 and 6. In contrast, cytokines produced by type 1 T-helper cells (Th1) - interleukins 2, 12 and IFNγ – are prevalent in the endometrium of women who had spontaneous miscarriages of unclear etiology in their medical history [18]. Besides, an increase of the IgM, IgA, and IgG levels is observed, which can lead to the rejection of the embryo.

## Material and Methods

Based on the above-given facts, screening and treatment of 70 patients of reproductive age diagnosed with infertility were carried out. All patients were included in the in vitro fertilization protocol and divided into two groups: the main group (n=40) and the control group (n=30). To diagnose the diseases of the reproductive tract, a standard clinical and laboratory screening of all patients was carried out including the description of quantitative and qualitative characteristics of vaginal discharge (color, consistency, odor); examination of the mucous covering of the vulva and vagina (color, lesions, presence of papillomata); microscopic examination of Gram-stained vaginal swabs; endocervical cultures and STIs diagnostics using polymerase chain reaction (PCR). The infectious process of the patients in the main group was treated using intravaginal autolymphocyte therapy (IALT) together with traditionally-accepted treatment schemes (etiotropic antibacterial and antifungal therapy). Traditional treatment methods were used in the control group, depending on the etiology of the development of infection. The patients were treated with IALT in the following way:

• after sampling 10-20 ml of venous blood, the lymphocytic suspension was prepared; after the cells were counted in the hemocytometer, their concentration was brought to 106–107 cells/ml;
• the received cells were re-suspended in a sterile isotonic normal saline solution and cultivated together with an immune-response modifier (Imunofan) for 3-4 hours at 37°C in an atmosphere containing 5% CO2 and 95% humidity;
• intravaginal introduction of autolymphocytes was carried out in a day hospital under gynecologist’s control; the suspension was introduced in 5.0 ml of physiological saline 1-2 times per week;
• the number of procedures was from 6 to 8, exposed for 40-60 minutes [19].

The treatment effectiveness was assessed in 12-14 days after the completion of therapy (first control) and 28-31 days (second control). The cure was acknowledged in case of the absence of complaints, clinical and laboratory indicators of an infectious process. Absolute absence of therapeutic effect was diagnosed in the case of patients that still had complaints, and the disease symptoms aggravated. For a comprehensive assessment of the therapy role in the correction of immune disorders, the immune status of patients before and after the treatment was studied in the framework of the research.

## Results

The examination of patients from the two groups showed that >50% of women suffered from one or more episodes of inflammatory diseases of the female reproductive tract. Moreover, with part of the examined females, a trend was identified towards a recurring and protracted course of an inflammatory process (42.5% in the main group and 46.7% in the control group), which can indicate disorders in the screening results of patients for STIs and the body’s defense system on local and systemic levels.

### Screening of patients for STIs

Agents causing urogenital infections detected using the PCR method are presented in Table 1. When testing the vaginal swabs, 11 (15.7%) patients had indicators of a fungal infection (per field of vision – spores and mycelium of yeast-like fungi). However, the microscopic examination did not detect indicators of yeast-like fungi in 5 (7.1%) patients with typical complaints of itching and burning in the vagina. The growth of Candida albicans in the amount of 104 CFU/ml was detected when assessing vaginal secretion cultures. In 9 (12.9%) patients, the culture tests detected the growth of associations of bacteria, and in these cases, the growth of lactobacilli was reduced sharply or was not detected at all. In 17% of patients, the so-called ‘key’ cells were detected in the smears – these are vaginal epithelial cells with small bacillary bacteria adsorbed in them. Notably, these patients did not have elevated white cells response (leucocytes – no more than 20 per field of vision). This microscopic picture was inherent for bacterial vaginosis associated with Gardnerella vaginalis.

**Table 1:** Patients’ screening results for STIs; n (%).

Indicator	Main group (40 patients)	Control group (30 patients)
**Chlamydia trachomatis**	3 (7.5%)	5 (16.6)
**Mycoplasma genitalium**	2 (5.0%)	3 (10.0%)
**Mycoplasma hominis**	2 (5.0%)	2 (6.7%)
**Ureaplasma urealiticum / Ureaplasma parvum**	8 (20.0%)	9 (30.0%)
**Gardnerella vaginalis**	7 (17.5%)	5 (16.6%)
**Candida albicans**	9 (22.5%)	7 (23.3%)
**Human papillomavirus (type 6, 11)**	1 (2.5%)	2 (6.7%)
**Human papillomavirus with a high cancerogenic risk**	8 (20.0%)	6 (20.0%)
**Herpes simplex viruses 1 and 2**	3 (7.5%)	2 (6.7%)
**Cytomegalovirus**	5 (12.5%)	3 (10.0%)

### Clinical presentations of an infectious process

The examination of clinical indicators of a vulvovaginal infection indicated that this pathology presented itself, mainly, by an increase in the amount of vaginal discharge (Table 2) and its quality. Particularly, 54.3% of patients complained about increased discharge, and the discharge was white or yellowish, rarely – with a shade of green, the consistency was homogenous, foamy, or thick. In 21.4% of cases, patients complained about itching or burning in the genital area; some pointed out that the intensity of these manifestations changed depending on the menstrual cycle phase. This clinical presentation was most often observed in the case of Candida, affecting mucous membranes, as well as if it was also combined with other infections, more rarely – with herpes virus infection. A major complaint of 24.3% of the examined patients was an unpleasant odor of the discharge, typical for patients with bacterial vaginitis (70.5%), and a complex form of vulvovaginitis (29.4%). 12.9% of patients in both groups complained about dysuria, which was more characteristic of comorbid pathology. Three (4.3%) patients suffered from dyspareunia. All the mentioned complaints were not specific. Additionally, 10 (25%) women in the main group and 6 (20%) in the control group did not have complaints, which may indicate a latent infection.

**Table 2:** Clinical presentations of an infectious process; n (%).

Indicator	Main group (40 patients)	Control group (30 patients)
**Abundant discharge**	22 (55.0%)	16 (53.3%)
**Itching and burning**	9 (22.5%)	6 (20.0%)
**Unpleasant odour of the discharge**	10 (25.0%)	7 (23.3%)
**Dysuria**	5 (12.5%)	4 (13.3%)
**Dyspareunia**	2 (5.0%)	1 (3.3%)
**Asymptomatic course of the process**	10 (25.0%)	6 (20.0%)

For this reason, despite the presence of subjective symptoms, all patients were thoroughly examined with an assessment of vaginal discharge and state of mucous membranes of vulva and vagina. Indicators of an inflammatory process (hyperemia, stromal swelling) during speculum examination were observed in 19 (27%) of the examined patients. Moreover, 11 (15.7%) of patients had typical indicators of cervicitis (hyperemia, hemophilia). In some patients (5.7%), these manifestations spread to the vulva. A conclusion can be drawn from the afore given data that in the case of genital inflammatory diseases there are various subjective and clinical presentations, predisposition to recurrence and chronicity of the process (44.3%), as well as combination of polymicrobial associations and presence of indolent disease forms with few symptoms, which complicates the diagnosis and treatment, especially in the preconception period. This dictates the need to develop a treatment algorithm aimed at the elimination of mechanisms launching an inflammatory process, which would also activate the body’s general and local defense systems. After the treatment, a control examination was carried out.

### Therapeutic effectiveness of traditional and comprehensive treatment

According to the results of the first control examination, the disappearance of clinical symptoms was stated for 36 (90%) patients of the main group and 22 (73.3 %) of the control group (Table 3). According to the results of a microscopic analysis of vaginal swabs during the first control examination, indicators of infection were not present in the main group - in 30 (75.0%) of patients and 18 (60.0%) in the control group. There was no effect from therapy in 4 (10.0%) women of the main group and 8 (26.6%) of the control group, which led to the second course of treatment. The data received indicate that a combination of traditional therapy with the use of autolymphocytes is credibly more effective than using only etiotropic therapy.

**Table 3:** Therapeutic effectiveness of traditional and comprehensive treatment; n (%).

Treatment Results	1^st^ control screening (in 10-14 days)		2^nd^ control screening (in 28-31 days)	
	Main group (40 patients)	Control group (30 patients)	Main group (40 patients)	Control group (30 patients)
**Cure**	30 (75.0%)*	18 (60.0%)	37 (92.5%)*	23 (76.66%)
**Improvement**	6 (15.0%)	4 (13.33%)	–	–
**Absence of effect**	4 (10.0%)*	8 (26.66%)	3 (7.5%)*	7 (23.33%)

* differences between groups are statistically significant (р<0.05)

It is worthy of noting that in the case of 3 patients (10%) in the control group, women developed a Candida infection or there was a change of the microbial agent with the emergence of resistance to the medications used, which made it necessary to prescribe a repeated course of treatment, and, consequently, the overall medicinal strain on the body increased. This refers not only to systemic medications but also to locally used drugs, long-term use of which can disrupt the normal biocenosis of the vagina, whereas comprehensive treatment demonstrated the absence of similar complications.

The results of the second control examination showed that including IALT in the treatment scheme enabled us to reduce the number of relapses of infectious processes - 7.5% in the main group vs. 23% in the control group, which can be explained by the fact that etiotropic therapy does not influence the restoration of local body defense abilities. Therefore, the inclusion of IALT in standard etiotropic treatment schemes made it possible to reliably reduce the frequency of complications of pharmacological therapy as well as the likelihood of recurrence of the infectious process.

## Discussion

It should be noted that an increase of the IgM level was detected in the prevailing number of patients at the moment of diagnosing an inflammatory process, which indicates a primary low specific response of the organism to a foreign agent. This process was accompanied by the following changes of the cell element of the immune system: enhanced activation of B cells, reduction of activity of T-helper cells, which facilitate the diversion to the production of IgG class antibodies instead of IgM. There was also a certain decrease of NK cells count as well as the number of monocytes and macrophage-type cells, which demonstrates the activation of the defense immunity element responsible for pathogen elimination from the organism. Repeated analysis of immunograms of patients that underwent traditional treatment showed the absence of dynamics of IgM levels and correlation of T cells subpopulations; in 10% of cases, there was also further growth of B cells, which may indicate a propensity for a protracted process. Comprehensive use of traditional therapy and IALT in the treatment of urogenital infections demonstrated an immune correcting action of the latter, representing itself by a decrease of IgM concentration, normalization of correlation of helpers and suppressors, moderate increase of NK cell count, monocytes and granulocytes in 28-31 days after completion of the treatment in more than half of patients of the main group [20, 21]. Consequently, the comprehensive use of IALT ensures the necessary immune-modulating effect alongside the correction of stable vaginal dysbiosis.

## Conclusion

Therefore, a comparative assessment of traditional therapy and extensive use of medications and IALT shows that in the first case, treatment is justified in the case of single or rare episodes of vulvovaginal infections, which is also economically beneficial. However, in the case of relapsing vulvovaginitis and mixed infections accompanied by disorders in the immune defense at different levels, the use of IALT in a comprehensive therapy can be assessed as advisable and pathogenetically substantiated. Comparative analysis of remote results of the treatment indicated an advantage of extensive use of autolymphocyte therapy in the treatment of infectious processes over the traditional methods. 87.5% of patients of the main group had no indicators of aggravation or relapsing of the infectious process for a period of six months, whereas this indicator amounted to 36.6% in the comparison group. This result can be explained by a polysystemic impact of autolymphocyte therapy on the organism, which improves the body’s resistance to infectious agents. In this way, etiopathogenetic substantiation of IALT use is its broad spectrum of action against various elements of the infectious process: direct impact on the pathogen, anti-inflammatory action, change of the metabolic activity and mobilization of reparation mechanisms, normalization of immune status indicators. Additionally, this method is safe; no side effects or allergic reactions have been found.

## Conflict of Interest

The authors confirm that there are no conflicts of interest.
